# Supplementary material for: Synthesis and Characterization of Cyclodextrin-Based Polyhemiaminal Composites with Enhanced Thermal Stability
Source: Polymers (Basel). 2022 Apr 11;14(8):1562. doi: 10.3390/polym14081562 (PMC9032068; doi:10.3390/polym14081562)
Supplement: Supplementary file 1 [file polymers-14-01562-s001.zip › polymers-1669233-supplementary.pdf]

polymers

Supplementary Materials

Synthesis and Characterization of Cyclodextrin-Based Polyhemiaminal Composites with Enhanced Thermal Stability

Hoque Mohammed Jabedul [1,*], Mitsuo Toda [2,*] and Nobuyuki Mase [1,2,3]

Methods: ^{1}H and ^{13}C NMR were recorded on a Bruker Advance Ultra shield (400.13 MHz for ^{1}H, 100 MHz for ^{13}C) instrument, Bellerica, USA. Data for ^{1}H NMR are reported as chemical shift (δ ppm), multiplicity (s = singlet, d = doublet, t = triplet, q = quartet, m = multiplet), coupling constants (Hz), integration. Data for ^{13}C NMR are reported as chemical shifts. TMS is used as an internal standard. All recorded NMR spectrums of the compounds were analyzed by TopSpin 3.5p17 software and all peaks positioning was speculated by ChemDraw professional software with regarding references. Polymerization monitoring initially required amount of diamine and α-CD were taken into the small flask and DMSO-d_6 was added. The reaction mixture was sonicated for 1 h then 4.5 equivalent amount of formalin was added to that and mixed thoroughly by stirring. An aliquot was transferred into an NMR tube and capped, which then heated at 60 °C preheated oil bath for 30 min. NMR was recorded with 64 times scanning for ^{1}H and 1024 times scanning for ^{13}C.

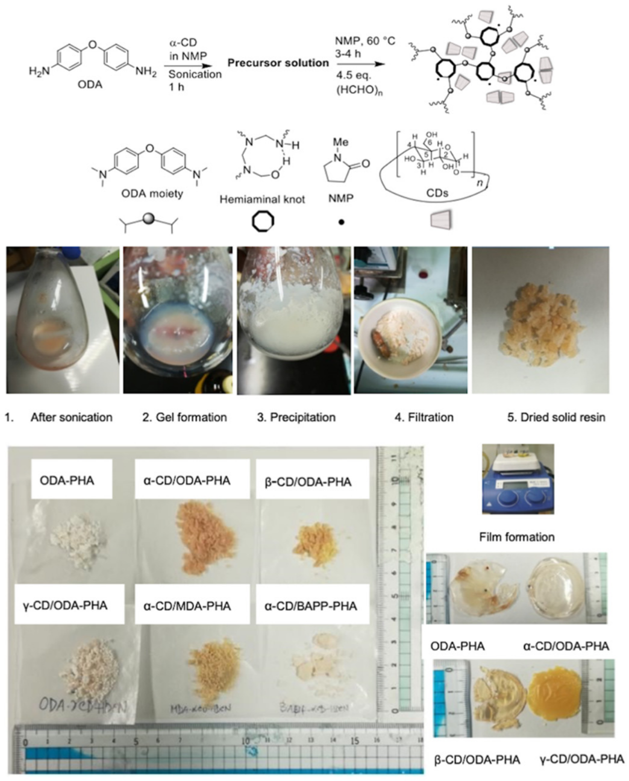

Scheme S1. Synthesis scheme with stepwise physical state monitoring, products specification, and film formation in an open bath at 60 °C for 18–24 h (for γ-CD/ODA-PHA film 48 h) inside the fume hood.

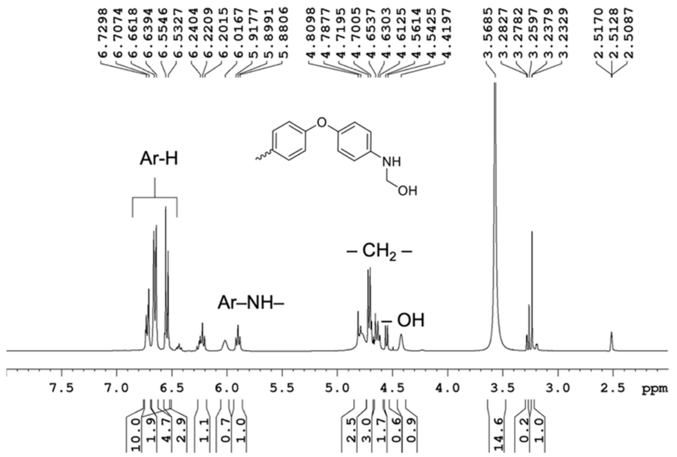
Ar-H
Ar–NH–
– CH2 –
– OH
ppm

(a)

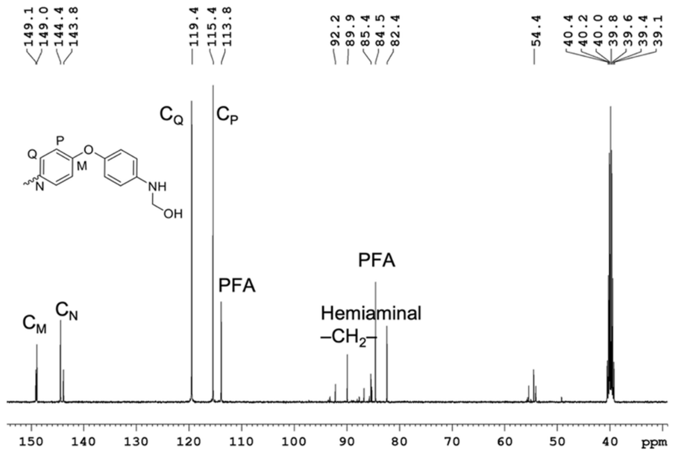
CQ
CP
PFA
CM
CN
Hemiaminal
–CH2–
PFA
ppm

(b)

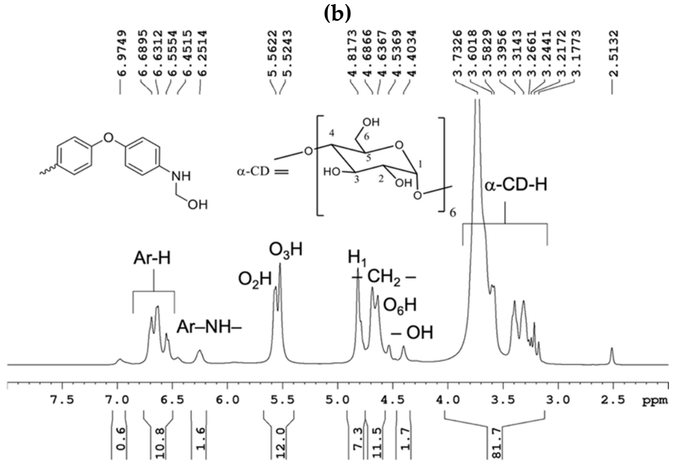
α-CD =
Ar-H
Ar–NH–
O2H
O3H
H1
– CH2 –
O6H
– OH
α-CD-H
ppm

(c)

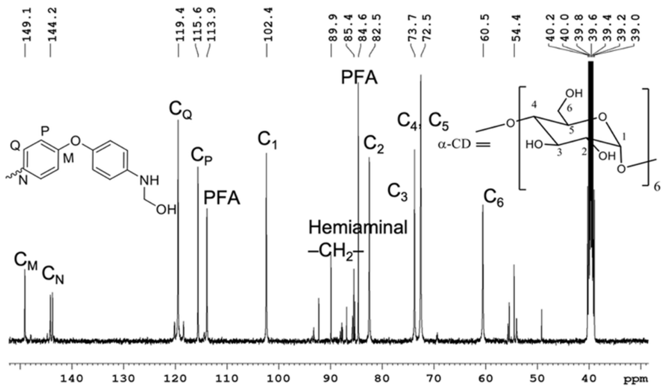

PFA
C_Q
C_1
C_4, C_5
α-CD =
C_P
C_2
PFA
C_3
C_6
Hemiaminal
$-CH_2-$
C_M
C_N
ppm

(d)

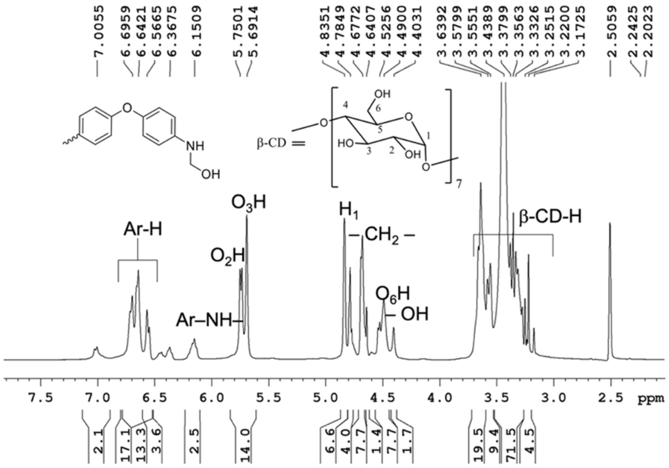

β-CD =
O_3H
H_1
Ar-H
$-CH_2-$
β-CD-H
O_2H
O_6H
Ar–NH–
– OH
ppm

(e)

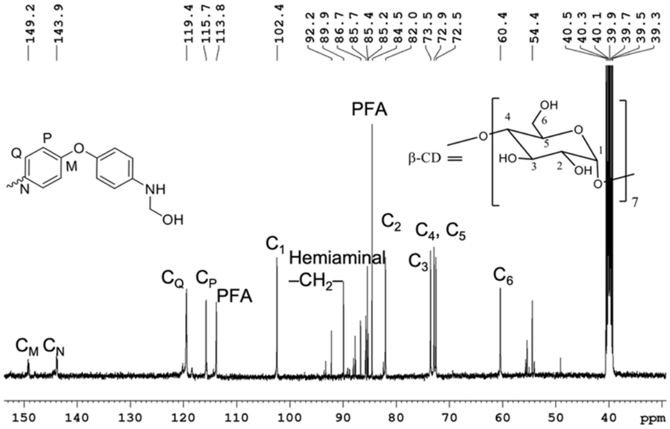

PFA
β-CD =
C_2
C_1
C_4, C_5
Hemiaminal
$-CH_2-$
C_3
C_Q
C_P
PFA
C_6
C_M
C_N
ppm

(f)

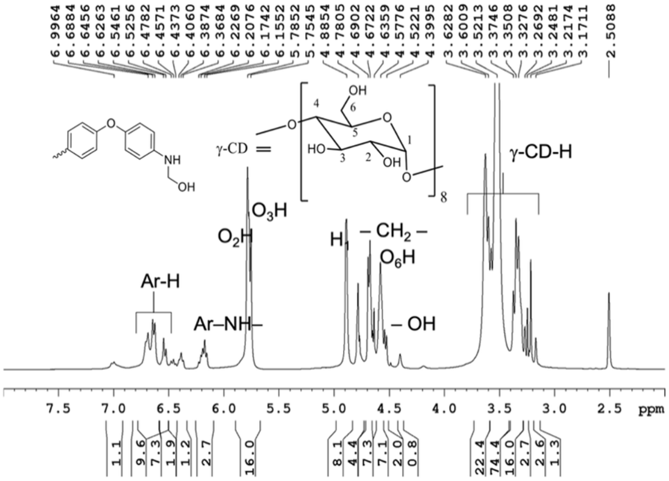
6.9964
6.6884
6.6456
6.6263
6.5461
6.5256
6.4782
6.4571
6.4373
6.4060
6.3874
6.3684
6.2269
6.2076
6.1742
6.1552
5.7852
5.7545
4.8854
4.7805
4.6902
4.6722
4.6359
4.5776
4.5221
4.3995
3.6282
3.6009
3.5213
3.3746
3.3508
3.3276
3.2692
3.2481
3.2174
3.1711
2.5088
γ-CD =
γ-CD-H
O_3H
O_2H
H_1
– CH_2 –
O_6H
Ar-H
Ar–NH–
– OH
ppm
1.1
9.6
7.3
1.9
1.2
2.7
16.0
8.1
4.4
7.3
7.1
2.0
0.8
22.4
74.4
16.0
2.7
2.6
1.3

(g)

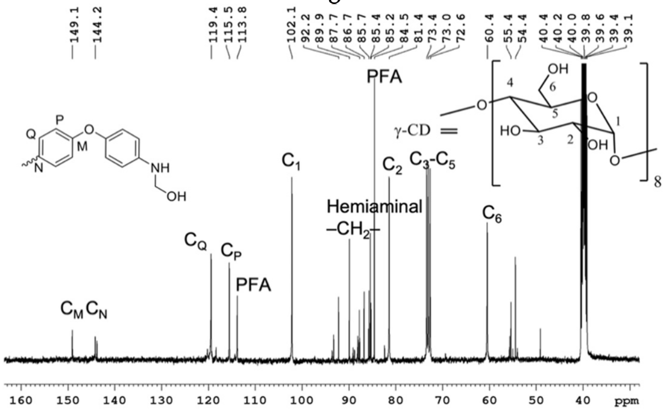
149.1
144.2
119.4
115.5
113.8
102.1
92.2
89.9
87.7
86.7
85.7
85.4
85.2
84.5
81.4
73.4
73.0
72.6
60.4
55.4
54.4
40.4
40.2
40.0
39.8
39.6
39.4
39.1
PFA
γ-CD =
C_1
C_2
C_3-C_5
Hemiaminal
$-CH_2-$
C_6
C_Q
C_P
PFA
C_M C_N
ppm

(h)

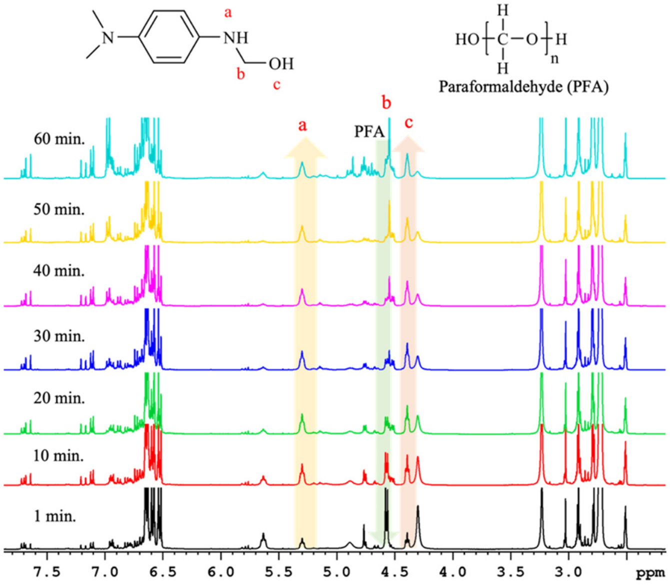
Paraformaldehyde (PFA)
60 min.
50 min.
40 min.
30 min.
20 min.
10 min.
1 min.
a
PFA
b
c
ppm

(i)

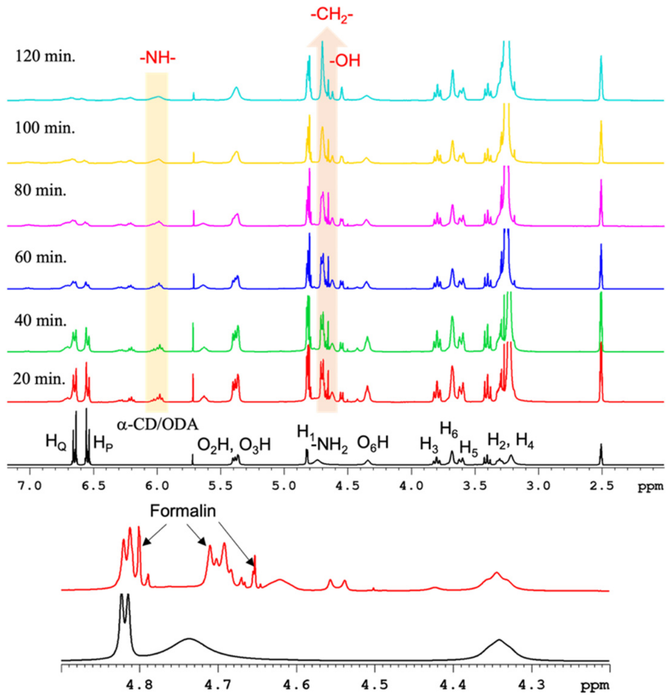

(j)

Figure S1. (**a**): ODA-PHA formation in DMSO-d_6 after 20 min reaction at 50–55 °C before gel formation. (**b**): ^{13}C NMR spectrum of ODA-PHA polymer in DMSO-d_6 after 20 min reaction at 50–55 °C before gel formation. (**c**): α-CD/ODA-PHA formation in DMSO-d_6 after 20 min reaction at 50–55 °C before gel formation. (**d**): α-CD/ODA-PHA formation in DMSO-d_6 after 20 min reaction at 50–55 °C before gel formation. (**e**): β-CD/ODA-PHA formation in DMSO-d_6 after 20 min reaction at 50–55 °C before gel formation. (**f**): β-CD/ODA-PHA formation in DMSO-d_6 after 20 min reaction at 50–55 °C before gel formation. (**g**): γ-CD/ODA-PHA formation in DMSO-d_6 after 20 min reaction at 50–55 °C before gel formation. (**h**): γ-CD/ODA-PHA formation in DMSO-d_6 after 20 min reaction at 50–55 °C before gel formation. (**i**): ^{1}H-NMR spectra of DMPD-hemiaminal bond formation at 55 °C NMR probe temperature up to 60 minutes after 10 minutes intervals. Hemiaminal -NH-, -CH_2-, and -OH peaks are denoted by a, b, and c respectively. (**j**): ^{1}H-NMR spectra of α-CD/ODA-hemiaminal bond formation at 55 °C NMR probe temperature up to 120 minutes after 10 minutes intervals. Hemiaminal peaks are assigned as follows -NH-, -CH_2-, and -OH.

^{1}H NMR (400 MHz, DMSO-d_6): δ (ppm) = Aromatic proton peaks appeared at 6.73–6.53. Other peaks are assigned as follows 5.90 (hemiaminal, Ar–NH–), 4.72 (hemiaminal, –CH_2–), 4.42 (hemiaminal, –OH).

ODA-PHA: δ (ppm) = 149.1 (C_M), 143.8 (C_N), 119.4 (C_Q), 115.4 (C_P). Apart from that, a hemiaminal (–CH_2–) peak at 89.9 ppm, unreacted PFA peaks at 113.8 ppm and 84.5 ppm were observed.

^{1}H NMR (400 MHz, DMSO-d_6): δ (ppm) = Aromatic proton peaks appeared at 6.97–6.45. Other peaks are assigned as follows 6.25 (hemiaminal, Ar–NH–), 5.56 (O_2H), 5.52 (O_3H), 4.82 (H_1), 4.69 (hemiaminal, –CH_2–),4.64 (O_6H), 4.40 (hemiaminal, –OH), 3.73–3.18 (H_2 to H_5).

^{13}C NMR (DMSO-d_6, 100 MHz): α-CD: δ (ppm) = 102.4 (C_1), 82.4 (C_2), 73.7 (C_3), 72.5 (C_4 and C_5), 60.5 (C_6).

ODA: δ (ppm) = 149.1 (C_M), 144.2 (C_N), 119.4 (C_Q), 115.6 (C_P). Apart from that, a hemiaminal (–CH_2–) peak at 89.9 ppm and an unreacted PFA peak at 113.9 ppm and 84.6 ppm were observed.

^{1}H NMR (400 MHz, DMSO-d_6): δ (ppm) = Aromatic proton peaks appeared at 7.01–6.57. Other peaks are assigned as follows 6.15 (hemiaminal, Ar–NH–), 5.75 (O_2H), 5.69

(O_3H), 4.84 (H_1), 4. (hemiaminal, –CH_2–), 4.49 (O_6H), 4.40 (hemiaminal, –OH), 3.64–3.25 (H_2 to H_5).

^{13}C NMR (DMSO-d_6, 100 MHz): β-CD: δ (ppm) = 102.4 (C_1), 82.0 (C_2), 73.5 (C_3), 72.9 (C_4), 72.5 (C_5), 60.4 (C_6).

ODA: δ (ppm) = 149.2 (C_M), 143.9 (C_N), 119.4 (C_Q), 115.7 (C_P). Apart from that, a hemiaminal (–CH_2–) peak at 89.9 ppm and an unreacted PFA peak at 113.8 ppm and 84.5 ppm were observed.

^1H NMR (400 MHz, DMSO-d_6): δ (ppm) = Aromatic proton peaks appeared at 6.69–6.55 ppm. Other peaks are assigned as follows 6.21 (hemiaminal, Ar–NH–), 5.79 (O_2H), 5.76 (O_3H), 4.89 (H_1), 4.64 (hemiaminal, –CH_2–), 4.52 (O_6H), 4.40 (hemiaminal, –OH), 3.63–3.33 (H_2 to H_5).

^{13}C NMR (DMSO-d_6, 100 MHz): γ-CD: δ (ppm) = 102.1 (C_1), 81.4 (C_2), 73.4 (C_3), 73.0 (C_4), 72.6 (C_5), 60.4 (C_6).

ODA: δ (ppm) = 149.1 (C_M), 144.2 (C_N), 119.4 (C_Q), 115.5 (C_P). Apart from that, a hemiaminal (–CH_2–) peak at 85.7 ppm and an unreacted PFA peak at 113.8 ppm were observed.

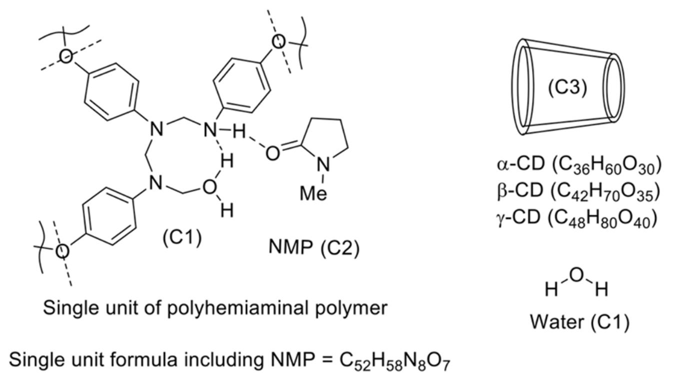

Figure S2. Hypothetical model based on elemental analysis. Following components were considered to calculate the percentage elemental composition (X_1) ODA component, (X_2) hemiaminal component, (X_3) NMP solvent, (*Y*) water, and (*Z*) CDs.

Hypothetical model based on possible PHA crosslink unit with CDs composition. From the previous report reveals that PHA polymer entrapped NMP solvent including water. We considered all the components including embedded NMP, water, and CDs composition.

The empirical formula was used to calculate percentage element composition:

$$C\% = \frac{12.011\,(X+nY+mZ)}{(MX+nMY+mMZ)} \times 100 \quad (1)$$

where, *n* (0, 1, and 2) is the number of moles water considered to calculate the elemental analysis based on experimental elemental analysis, *m* (0.5 and 1) is the number of moles CDs used to prepare composite polymer, *M* is the molecular weight of each component.

Table S1. Elemental composition (E.C) analysis data for ODA-PHA (three components consideration) and CDs/ODA-PHA composites (four components consideration) according to (Figure S2). [1] E.C obtained from elemental analysis data. [2] Theoretical calculations based on the above-mentioned formulas by considering possible components of polymers along with the bound solvent contribution.

Compound	E.C ratio of (C/H/N/O) % [1]	Calculated E.C ratio of (C/H/N/O) % Based on Regarding Formula [2]
ODA-PHA	65.9/6.1/11.4/16.6	66.2/6.6/11.9/15.3 ($C_{52}H_{62}N_8O_9$)
α-CD/ODA-PHA (ODA : α-CD/ 1:1)	49.8/6.4/4.3/39.5	51.6/6.4/3.9/38.2 ($C_{124}H_{182}N_8O_{69}$)

α-CD/ODA-PHA (ODA : α-CD/ 1:0.5)	54.0/6.7/7.1/32.2	55.2/6.4/5.9/32.6 ($C_{88}H_{122}N_8O_{39}$)
β-CD/ODA-PHA	55.3/6.8/6.7/31.2	55.3/6.3/5.5/32.9 ($C_{94}H_{128}N_8O_{42}$)
γ-CD/ODA-PHA	54.7/6.9/6.2/32.2	54.5/6.3/5.1/34.1 ($C_{100}H_{138}N_8O_{47}$)
α-CD/MDA-PHA	57.1/6.9/7.1/30.8	57.2/6.8/5.9/30.2 ($C_{91}H_{128}N_8O_{36}$)
α-CD/BAPP-PHA	55.9/7.0/6.3/30.8	62.7/6.5/4.4/26.4 ($C_{133}H_{164}N_8O_{42}$)

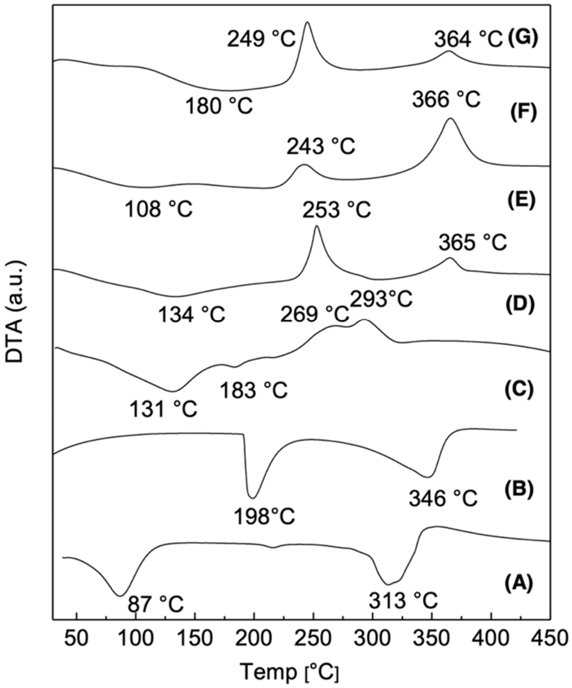

Figure S3. DTA thermograms of (A) α-CD, (B) ODA-PHA, (C) α-CD/ODA-PHA, (D) β-CD/ODA-PHA, and (E) γ-CD/ODA-PHA.

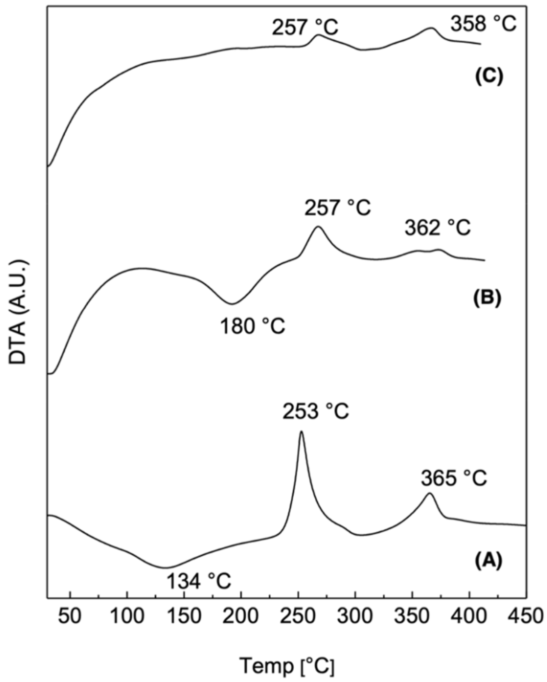

Figure S4. DTA thermograms of three different composites (A) α-CD/ODA-PHA, (B) α-CD/MDA-PHA, and (C) α-CD/BAPP-PHA) synthesized from three different diamines.

Table S2. Char yield at three decomposition temperatures including CDs (first decomposition temperature), ODA-PHA (second decomposition), and CDs/ODA-PHA composites (third decomposition temperature). Char yield at three decomposition temperatures was calculated from the remaining weight of the char in the raw data of the TGA analysis.

Components	Char yield at 1st Decomposition Temperature	Char yield at 2nd Decomposition Temperature	Char yield at 3rd Decomposition Temperature
ODA-PHA	-	65.5 (293 °C)	41.1 (364 °C)
α-CD/ODA-PHA	67.6 (253 °C)	61.8 (293 °C)	39.6 (365 °C)
β-CD/ODA-PHA	82.7 (242 °C)	75.0 (293 °C)	39.8 (365 °C)
γ-CD/ODA-PHA	79.0 (245 °C)	71.0 (293 °C)	42.9 (366 °C)

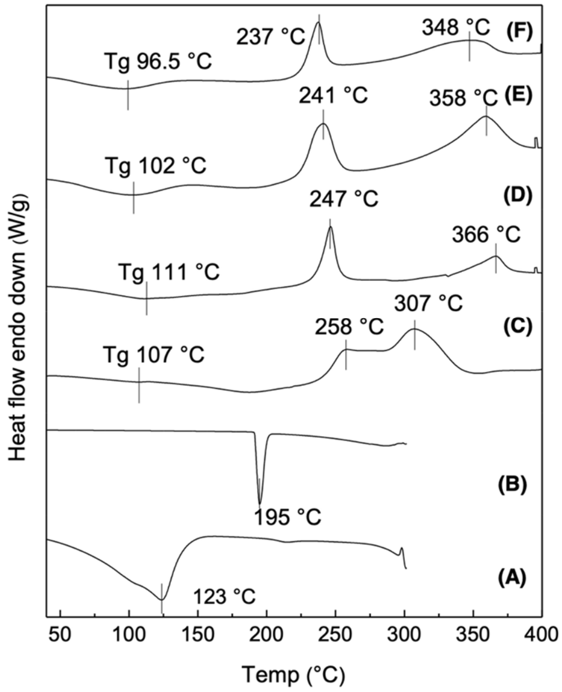

Figure S5. DSC thermograms of (A) α-CD, (B) ODA-PHA, (C) α-CD/ODA-PHA, (D) β-CD/ODA-PHA, and (E) γ-CD/ODA-PHA.

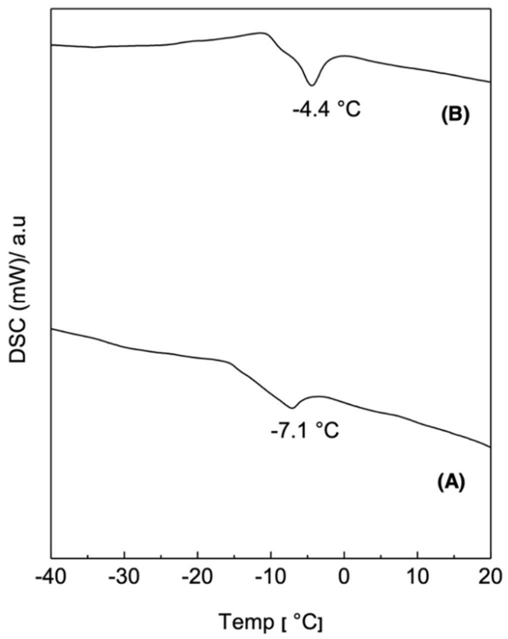

Figure S5. a: DSC thermograms of both (A) α-CD/ODA-PHA and (B) ODA-PHA at temperature range -40 to 40 °C by using liquid Nitrogen.

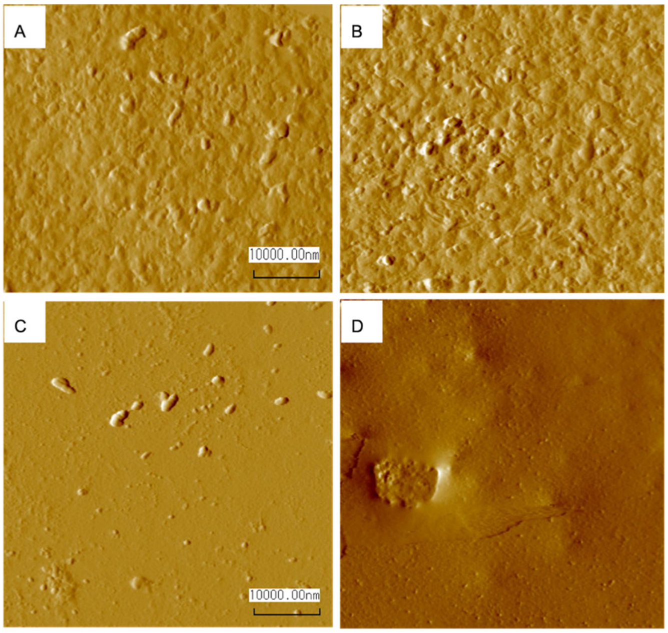

Figure S6. AFM images of ODA-PHA (A and B) and α-CD/ODA-PHA (C and D) films prepared at 60 °C.

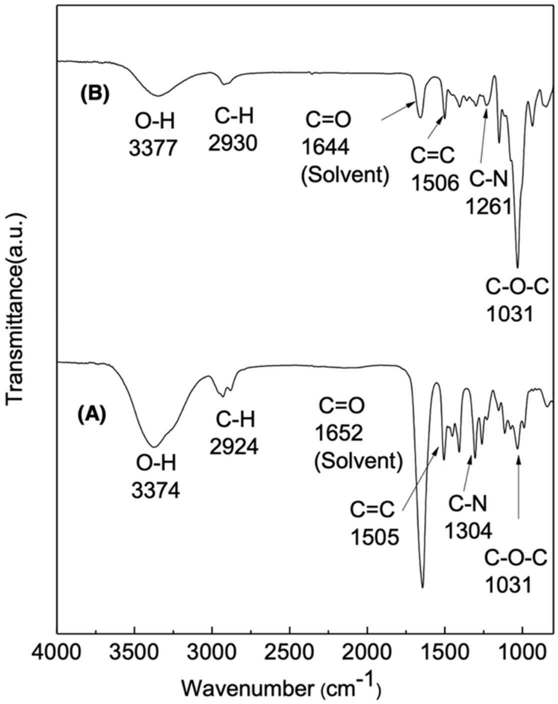

Figure S7. FTIR spectra of both (A) α-CD/ODA-PHA film (B) dissolved portion of α-CD/ODA-PHA film in NMP/LiBr (8–9 w/w%) solvent.

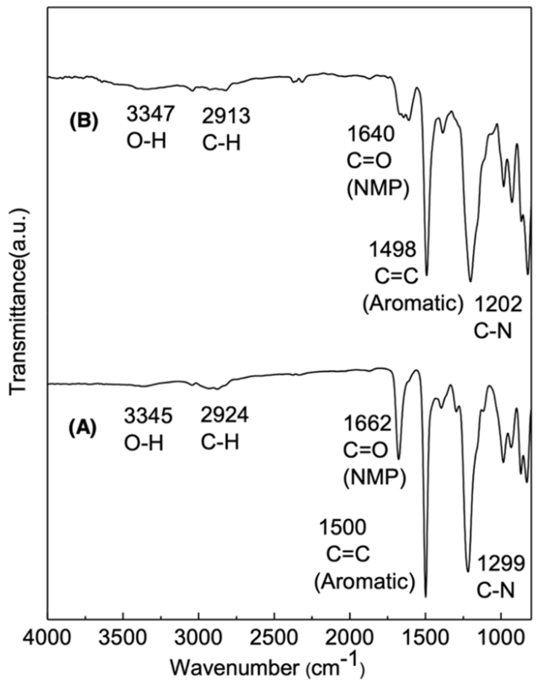

Figure S7. a: FTIR spectrum of both (A) ODA-PHA film (B) Undissolved portion of α-CD/ODA-PHA films in NMP/LiBr (8–9 w/w%) solvent.

Diamine recovery test:

Approximately 0.2 g of α-CD/ODA-PHA composite was taken in the vial. Added 6 ml 1N H_2SO_4, the polymer was disintegrated to obtain a clear solution within 5 min. Added Na_2CO_3 1M solution dropwise, bubbling of CO_2 observed, with the rising of pH precipitation start to appear. After maintaining pH around 7–8 precipitation was completed, and bubbling stopped. The precipitate was filtered and collected then dried under a vacuum. NMR and FTIR were recorded and compared with authentic ODA (shown in Figures S9 and S10).

Later, approximately 0.08 g of ODA-PHA and CDs/ODA-PHA composite films were taken in the watch glass. Added 1 ml solution in each plate and allowed to disintegrate. Required time was recorded for each of the specimens (Figure S8).

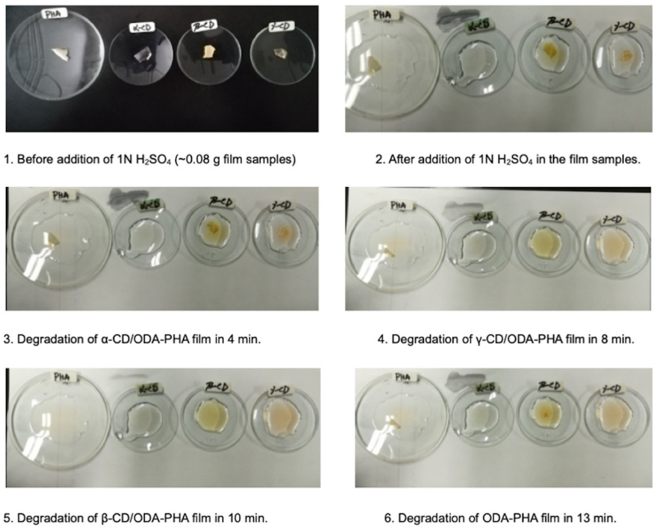

Figure S8. Time required for the acid digestion of ODA-PHA and CDs/ODA-PHA composite films in 1N H_2SO_4.

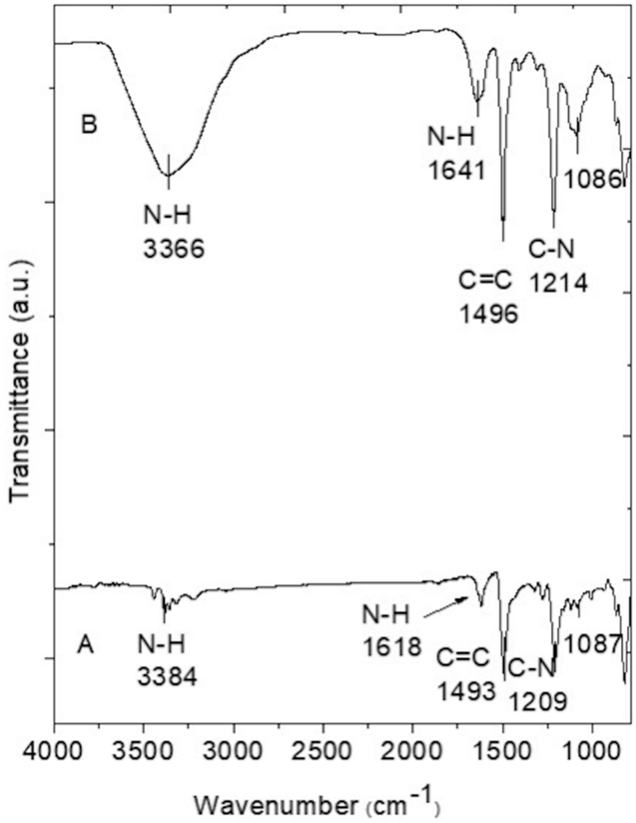

Figure S9. FTIR spectra of both pure ODA and recycled ODA.

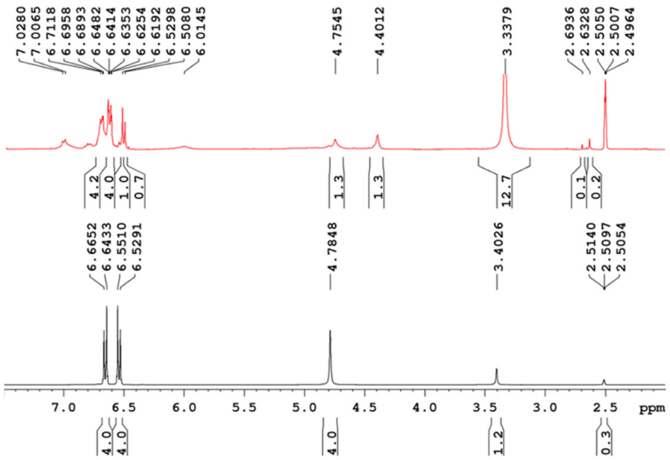

Figure S10. Overlapped ^{1}H NMR spectra of both ODA (black spectrum) and recycled ODA (red spectrum) in DMSO-d_6.
